# *Amorpha fruticosa* – A Noxious Invasive Alien Plant in Europe or a Medicinal Plant against Metabolic Disease?

**DOI:** 10.3389/fphar.2017.00333

**Published:** 2017-06-08

**Authors:** Ekaterina Kozuharova, Adam Matkowski, Dorota Woźniak, Rumiana Simeonova, Zheko Naychov, Clemens Malainer, Andrei Mocan, Seyed M. Nabavi, Atanas G. Atanasov

**Affiliations:** ^1^Department of Pharmacognosy, Faculty of Pharmacy, Medical University of SofiaSofia, Bulgaria; ^2^Department of Pharmaceutical Biology with Botanical Garden of Medicinal PlantsMedical University of Wroclaw, Poland; ^3^Department of Pharmacology, Pharmacotherapy and Toxicology, Faculty of Pharmacy, Medical University of SofiaSofia, Bulgaria; ^4^Sofia University St. Kliment Ohridski, Faculty of Medicine, Department of Surgery, Obstetrics and Gynecology, Division of Cardiac Surgery, University Hospital LozenetzSofia, Bulgaria; ^5^Independent ResearcherVienna, Austria; ^6^Department of Pharmaceutical Botany, Iuliu Haţieganu University of Medicine and PharmacyCluj-Napoca, Romania; ^7^ICHAT and Institute for Life Sciences, University of Agricultural Sciences and Veterinary MedicineCluj-Napoca, Romania; ^8^Applied Biotechnology Research Center, Baqiyatallah University of Medical SciencesTehran, Iran; ^9^Institute of Genetics and Animal Breeding, Polish Academy of SciencesJastrzebiec, Poland; ^10^Department of Pharmacognosy, University of ViennaVienna, Austria; ^11^Department of Vascular Biology and Thrombosis Research, Center for Physiology and Pharmacology, Medical University of ViennaVienna, Austria

**Keywords:** plant chemical constituents, diabetes, metabolic syndrome, bioactive compounds, plant secondary metabolites, rotenoids

## Abstract

*Amorpha fruticosa* L. (Fabaceae) is a shrub native to North America which has been cultivated mainly for its ornamental features, honey plant value and protective properties against soil erosion. It is registered amongst the most noxious invasive species in Europe. However, a growing body of scientific literature also points to the therapeutic potential of its chemical constituents. Due to the fact that *A. fruticosa* is an aggressive invasive species, it can provide an abundant and cheap resource of plant chemical constituents which can be utilized for therapeutic purposes. Additionally, exploitation of the biomass for medicinal use might contribute to relieving the destructive impact of this species on natural habitats. The aim of this review is to provide a comprehensive summary and systematize the state-of-the-art in the knowledge of the phytochemical composition and the potential of *A. fruticosa* in disease treatment and prevention, with especial emphasis on diabetes and metabolic syndrome. Also reviewed are aspects related to potential toxicity of *A. fruticosa* which has not yet been systematically evaluated in human subjects.

## Introduction

*Amorpha fruticosa* L. (Fabaceae) is known by several common names, *viz.* false indigo-bush, desert false indigo, and bastard indigobush, which refer to its traditional use as a dye source. The plant is a shrub native to North America – contiguous United States, northern Mexico, and south-eastern Canada (Wilbur, 1975; USDA, NRCS, 2009; Anonymous, 2011). A mature plant has a broad crown with 1–10 stems growing to a height of 1.0–3.5 m and it is highly variable in morphology. The morphological variety of the plant is reflected by the fact that the species’ currently accepted name has at least 16 synonyms (DeHaan et al., 2006). The leaves are compound, odd-pinnate, 10–28 cm long with 9–21 leaflets that are 2–4 cm long and 1–2 cm wide. In the northern hemisphere, *A. fruticosa* blooms May and June with scented flowers that are purplish blue with orange anthers and occur in upright spikes. Linnaeus called this plant *Amorpha* because the flower has only a single petal (flag), while the other four petals that are normally present in legumes are entirely missing (Austin, 2004). The flowers are followed by fruits which mature in July and August. The fruits are short, smooth or hairy, glandular legumes containing one or two smooth brownish seeds (Dirr, 1997; DeHaan et al., 2006). The rich nectar production of these flowers with ten protruding stamens with yellow anthers makes false indigo, a highly appreciated honey plant and important food source for bees, both in its native range and in the invaded territories (Pellett, 1920; Kulinčević, 1959; Stubbs et al., 1992; Oddo et al., 2004; Stefanic et al., 2004; Tucak et al., 2007; Tuell et al., 2008; Grozeva and Budakov, 2010; Grozeva, 2011; Dimou et al., 2014; Hong et al., 2016).

*Amorpha fruticosa* became popular in Europe as ornamental plant in the early 1700s (Huxley, 1992; Austin, 2004). Its role as a honey plant also contributed to its cultivation (Jablonski and Koltowski, 2001). Additionally, it was planted to stabilize the soil (especially on railway embankments) due to its protective role against erosion provided by an extensive root system (Van Dersal et al., 1938; Bowie, 1982; Brigić et al., 2014). As a result of all these human activities *A. fruticosa* is registered among the worst Alien Invasive Species Inventories for Europe (DAISIE, 2009) and the detrimental effects of the plant on local biospheres have been investigated in several case studies (Szigetvári, 2002; Deák, 2005; Muranaka et al., 2005; Protopopova et al., 2006; DAISIE, 2009; Sărăţeanu, 2010; Petrova et al., 2012). *A. fruticosa* can also tolerate dry soils, but it is most abundant along river banks and roads and the edges of flooded forests. The plant grows well in medium to wet, well-drained, soils in full sun to light shade and is tolerant of occasional flooding. It has well-developed roots and is relatively wind-tolerant. It may spread by self-seeding and/or suckers to form thickets (Freeman and Schofield, 1991). This high tolerance of various habitat conditions and potent propagation ability promotes the aggressive invasive behavior of *A. fruticosa* outside of its native range. *A. fruticosa* is especially successful in colonizing degraded habitats (e.g., areas where agriculture or grazing has been abandoned), but it also invades natural plant communities, where it competes with native vegetation leading to a considerable increase in activity, density, and abundance of soil invertebrates, but at the same time it massively decreases species diversity (Brigić et al., 2014). Interestingly, the invasiveness of *A. fruticosa* has also been attributed to its allelopathic potential in terms of a so-called juglone index (Szabo, 1999) that was found to be highest in a study comparing fifteen invasive plant species occurring in Hungary (Csiszár, 2009; Csiszár et al., 2013). *A. fruticosa* invasion was observed to considerably affect carabid beetle species composition although these insects are known to be only indirectly related to plant composition (Brigić et al., 2014). Often the organisms that are predators on the invasive plants in the natural habitats are not present in the newly invaded habitats. Thus, the control of the populations is reduced which promotes the distribution of the invasive plants and jeopardizes the ecological balance. In the case of *A. fruticosa* the North American bruchid beetle *Acanthoscelides pallidipennis* (Motschulsky), the larvae of which feed in seeds, has been found in some of the invaded territories (Kyushu Island, Japan) and it could help to re-establish ecological balance. However, the effectiveness of such seed predators as natural enemies of invasive plant species is controversial (Tuda et al., 2001), and *A. fruticosa* remains amongst the most dangerous invasive species in Europe (DAISIE, 2009; Sărăţeanu, 2010; Petrova et al., 2012). At the same time, many examples reveal the effective destructive power of mankind when plants are used commercially. Exploitation of the populations of invasive plant species for medicinal purposes may be regarded as regulation ecosystem services and part of a sustainable development strategy. Such a strategy could contribute to balance the natural ecosystems and preserve biodiversity.

One of the quite promising medical applications of *A. fruticosa* is against diabetic complications. Diabetes is a serious, chronic disease caused by either insufficient insulin production from the pancreas (type 1), or when the body cannot effectively utilize the insulin it produces (type 2) (World Health Organization [WHO], 1999). Diabetes is one of the most important public health problems of our time, and its prevalence has been increasing over the past few decades. According to the World Health Organisation reports, the global prevalence of diabetes has doubled since 1980, rising from 4.7 to 8.5% in the adult population (World Health Organization [WHO], 2016). Diabetes leads to severe complications including diabetic neuropathy, diabetic micro and macro angiopathy, diabetic nephropathy and diabetic retinopathy. Further long-term complications with diabetes include cardiovascular disease, leg amputations, stroke, chronic renal failure, vision loss, and nerve damage.

Diabetic retinopathy is responsible for about 2.6% of blindness (Bourne et al., 2013). At least 80% of the end stage renal disease is caused by diabetes, hypertension or a combination of them, while the proportion attributed solely to diabetes ranges between 12% and 55% (United States Renal Data System, 2014). The risk of cardiovascular disease development elevates with rising fasting plasma glucose levels, even at lower values than those that meet the criteria for a diabetes diagnosis (Danaei et al., 2006; Singh et al., 2013). Amputations among the people with diabetes are typically 10 to 20 times more frequent than among the non-diabetic population (Moxey et al., 2011).

Diabetes directly caused 1.5 million deaths in 2012, and 2.2 million people died from additional complications, yielding 3.7 million deaths in a single year from a single disease. Forty-three percent of them occurred before the age of 70 (World Health Organization [WHO], 2016).

Therefore, diabetes is among the chief priorities in most of the health systems around the world. While type 1 diabetes is not preventable with current medical knowledge, there are different approaches to prevent type 2, and to minimize the complications and the premature death caused by all types of diabetes.

The vast majority of the cases with diabetes are type 2 (World Health Organization [WHO], 1999). There are several known risk factors that contribute to developing type 2 diabetes such as ethnicity, family history, smoking, older age etc. but the most important of them is excess body fat (obesity) (GBD 2013 Risk Factors Collaborators, 2015).

According to the estimations, the direct cost of diabetes to the world is more than 827 billion USD per year (Seuring et al., 2015; NCD Risk Factor Collaboration (NCD-RisC), 2016).

Metabolic syndrome is defined as a condition characterized by a variety of diagnostic criteria, most important of which are obesity, dyslipidaemia, type 2 diabetes and arterial hypertension. All of them contribute to an elevated risk of cardiovascular morbidity and mortality. Several different diagnostic sets of criteria exist: from the World Health Organization (Alberti and Zimmet, 1998), from the International Diabetes Federation [IDF] (2015), from the European Group for the study of Insulin Resistance (Balkau and Charles, 1999), the National Cholesterol Education Programme Adult Treatment Panel III (Expert Panel on Detection, Evaluation, and Treatment of High Blood Cholesterol in Adults, 2001), as well as from the American Association of Clinical Endocrinologists (Einhorn et al., 2003).

The cardiovascular risk increases continuously with the number of the syndrome components present (Andreadis et al., 2007), and the cumulative risk of the concurrent factors is greater than that of the individual risk factors alone (Reilly and Rader, 2003).

Plants have been a continuous source of therapeutic agents historically, and still today represent a valuable pool for the discovery and development of new therapeutics in general (Atanasov et al., 2015), as well as in the context of cardiovascular and metabolic disease in particular (Waltenberger et al., 2016).

Due to the fact that *Amorpha fruticosa* is a successful aggressive invasive species, it could provide a vast and cheap resource of plant chemical constituents which can be utilized for remedial purposes. Additionally, problems which this plant causes to the natural habitats in many European countries could be alleviated. The aim of this study is to review the plant chemical constituents and the potential of *Amorpha fruticosa* against diabetes and metabolic syndrome. In the context of safety in a possible medical application, considerations regarding a potential toxicity of *A. fruticosa* are also discussed.

## Ethnobotanical use of *Amorpha fruticosa*

Native Americans of the Great Plains employed several of the more common *Amorpha* species for a variety of uses. *Amorpha fruticosa* was used for bedding material, horse feed, arrow shafts, the stems were arranged on the ground to create a clean surface on which to put butchered meat, and name “false indigo” is related to the application of the plant as a blue dye (Hoffman, 1891; Gilmore, 1913, 1919; Smith, 1928; Vestal and Schultes, 1939; Munson, 1981; Kindscher and Noguera, 2002; Austin, 2004; Straub, 2010). For medicinal purposes such as stomach pain, intestinal worms, eczema, neuralgia, and rheumatism, the related species *A. canescens* was used and its powdered leaves were applied to wounds (Hoffman, 1891; Gilmore, 1913, 1919; Smith, 1928, Straub, 2010). Moreover, reports for medicinal use of *Amorpha fruticosa* are also available: The Seminoles used infusion from leaves and stems as a general tonic and also against rheumatism and chronic sickness together with other plants; the Omaha used the plant to cure wounds (Munson, 1981; Austin, 2004).

## Phytochemical Constituents of *Amorpha fruticosa*

**FIGURE 1:**
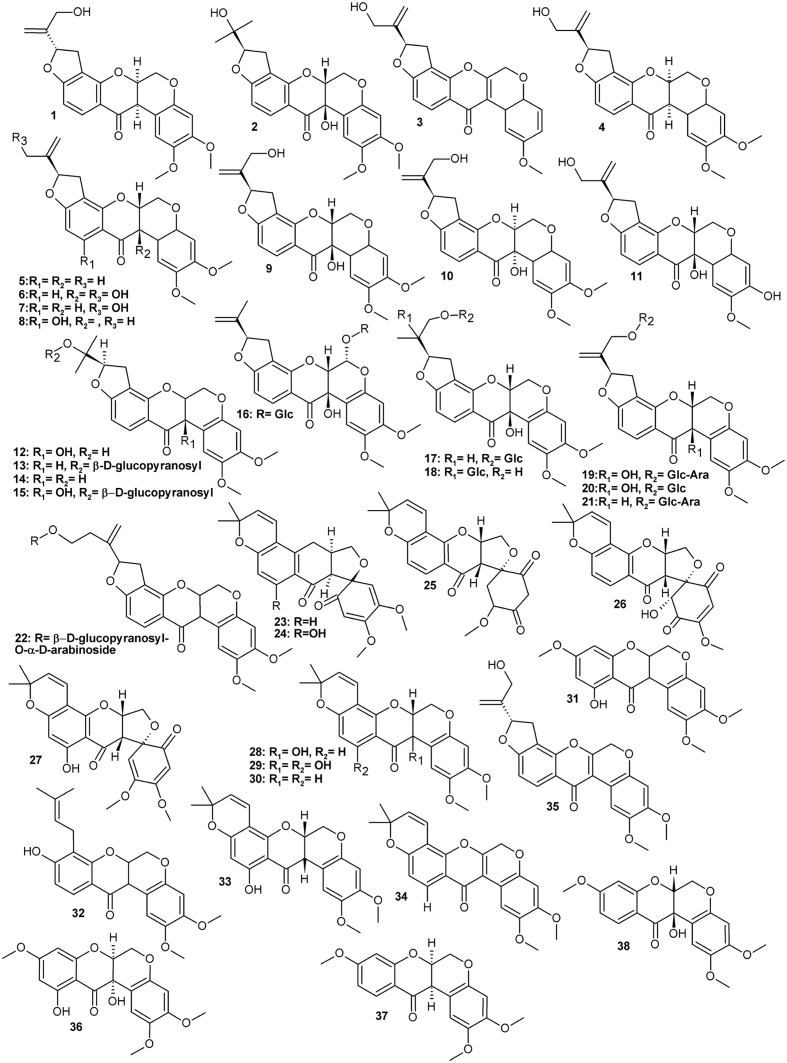
Rotenoids.

**FIGURE 2:**
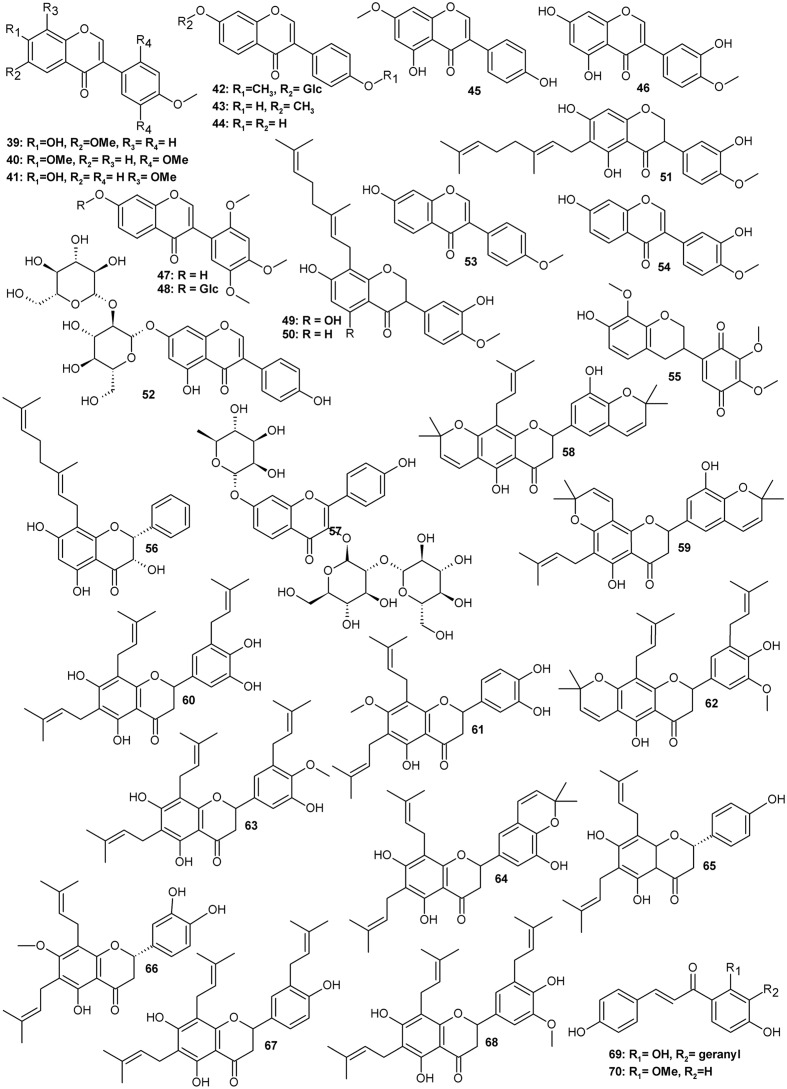
Flavonoids.

Typically for a leguminous plant, *Amorpha* contains a set of family marker classes such as isoflavonoids and their derivatives called rotenoids (**Figure 1** and **Table 1**), which result from the formation of an additional oxygen ring between rings B and C of the isoflavone skeleton (Crombie and Whiting, 1998; Hegnauer, 2001). Like many other specialized metabolites, flavonoids (**Figure 2** and **Table 2**) and rotenoids undergo further structural modifications after biosynthesis, which include prenylation, esterification, methylation, and addition of other various moieties.

**Table 1:** List of rotenoids isolated from various parts of *Amorpha fruticosa* plant.

				Contents or obtained
Compound		CAS Registry Number	Plant material	amount mg/g dry weight
**Rotenoids**				
1	Amorphigenin (8′ hydroxyrotenone)	4208-09-7	L, S, R	0.65
2	12β-Hydroxyamorphigenin	85042-77-9	A	n.a
3	6a,12a-Dehydro-3-*O*-demethylamorphigenin	not assigned	F	n.a.
4	(6aR,12aR,5′R)-Amorphigenin	stereoisomer of 1	F	0.05
5	Rotenone	83-79-4	F	0.01
6	Dalbinol	41993-79-7	F, R	0.745
7	Rotenolone	509-96-6	F	n.a
8	11-Hydroxyrotenone/(-)sumatrol	82-10-0	F	n.a.
9	6a*R*, 12a*R*-dalbinol (same as 6)	41993-79-7	S	1.49
10	6a*S*, 12a*S*-Dalbinol	stereoisomer of 6	S	0.02
11	3-*O*-Demethyldalbinol	stereoisomer of 98619-30-8	F	n.a.
12	12a-Hydroxydalpanol	85042-77-9	A, S	0.13
13	6′-*O*-β-D-Glucopyranosyldalpanol	52059-86-6	A	n.a.
14	Dalpanol	30462-22-7	F	0.02
15	6′-*O*-β-D-Glucopyranosyl-12a-hydroxydalpanol		F	n.a.
16	Amorphaside A	1703757-00-9	S	0.62
17	Amorphaside B	1703757-01-0	S	0.02
18	Amorphaside C	1703757-02-1	S	0.09
19	Amorphaside D	82873-12-9	S	0.86
20	Dalbin	68401-03-6	S	0.41
21	Amorphin	4207-90-3	S	0.26
22	Benzopyran-12-one,1,4,10,11-tetrahydro-6′-[8′-(hydroxymethyl)ethenyl]-2,3-dimethoxy-8′-*O*-β-D-glucopyranosyl-*O*-α-D-arabinoside	an enantiomer of amorphin,	A	n.a.
23	Amorphispironone	139006-28-3	L, F, S, T	0.03
24	1a*R*,6a*S*,12a*R*-11-Hydroxyamorphispironone	n.a.	L, T	
25	Amorphispironone B	n.a	F	0.06
26	Amorphispironone C		F	0.045
27	Hydroxyamorphispironone		F	0.21
28	Tephrosin	76-80-2	L, T	n.a
29	11-Hydroxytephrosin	72458-85-6	L, T	n.a.
30	(-)Deguelin	522-17-8	L, F, T	0.036
31	Sermundone	41630-82-4	F	0.05
32	Rot-2′-enonic acid	70191-71-8	F	0.009
33	α-Toxicarol	82-09-7	F	0.014
34	6a,12a-Dehydrodeguelin	3466-23-7	F	n.a
35	6a,12a-Dehydroamorphigenin	29444-01-7	R	n.a.
36	6-Deoxyclitoriacetal	146163-05-5	F	n.a.
37	Mundoserone	3564-85-0	F	n.a
38	12a-Hydroxymunduserone	66280-24-8	S	0.2

*According to Acree et al. (1943), Konoshima et al. (1993), Terada et al. (1993), Ohyama et al. (1998), Lee et al. (2006a), Dat et al. (2008), Diao et al. (2009), Kim et al. (2011), Wu et al. (2015, 2016), Muharini et al. (2017). Plant organs: A, aerial parts; L, leaves; F, fruits; S, seeds; R, roots; T, twigs*.

**Table 2:** Isoflavonoids (non-rotenoid) and other flavonoids isolated from various parts of *Amorpha fruticosa* plant.

				Contents or obtained
Compound		CAS Registry Number	Plant material	amount mg/g dry weight
**Other isoflavonoids**				
39	Afrormosin	550-79-8	A	n.a
40	7,2′,4′,5′-Tetramethoxyisoflavone	4253-02-5	A	n.a.
41	8-*O*-Methylretusin	37816-20-9	A	n.a
42	Ononin	486-62-4	S	0.02
43	Isoformonentin	486-63-5	S	0.01
44	Daidzein	486-66-8	S	0.02
45	Prunetin	552-59-0	S	0.01
46	Pratensein	2284-31-3	S	0.01
47	7-Hydroxy-2′,4′,5′-trimethoxyisoflavone	29096-94-4	S	0.01
48	7-*O*-β-D-Glucopyranosyl-7-hydroxy-2′,4′,5′-trimethoxyisoflavone	Not assigned	S	0.4
49	8-Geranyl-5,7,3′-trihydroxy-4′-methoxyisoflavone	Not assigned	F	0.25
50	8-Geranyl-7,3′-dihydroxy-4′-methoxyisoflavone	Not assigned	F	
51	6-Geranyl-5,7,3′-trihydroxy-4′-methoxyisoflavone	Not assigned	F	0.087
52	Daidzein 7-*O*-β-D-glucopyranosyl-(1→2)-B-D-glucopyranoside	Not assigned	F	0.036
53	Formononetin	485-72-3	R	n.a
54	Calycosin	20575-57-9	R	n.a.
55	Amorphaquinone	70283-29-3	R	n.a.
**Flavones**				
56	3,5,7-Trihydroxy-8-C-geranyl-flavanone	Not assigned	F	0.025
57	resokaempferol 3-*O*-B-Dglucopyranosyl-(1→2)-β-D-Glucopyranoside-7-*O*-L-rhamnopyranoside	Not assigned	F	0.01
**Prenylflavanones from roots**				
58	Amorin	119347-09-0	R	n.a
59	Isoamorin	119347-11-4	R	n.a.
60	Amorisin	83474-70-8	R	n.a.
61	Amoradicin	83677-03-6	R	n.a
62	Amoricin	119347-01-2	R	n.a.
63	Isoamoritin	212069-24-4	R	n.a.
64	Amorinin	83677-05-8	R	n.a
65	Amoradin	119347-05-6	R	n.a.
66	Amoradinin	94927-38-5	R	n.a.
67	Amorilin	83474-69-5	R	n.a
68	Amoritin	83474-68-4	R	n.a.
**Chalcones**				
69	Xanthoangelol	62949-76-2	L, F, T	0.023
70	2′-Methoxyisoliquiritigenin	112408-67-0	L, T	n.a.

*According to Acree et al. (1943), Rózsa et al. (1982, 1984, 1988), Konoshima et al. (1993), Terada et al. (1993), Ohyama et al. (1998), Lee et al. (2006b), Dat et al. (2008), Diao et al. (2009), Kim et al. (2011), Wu et al. (2015, 2016), Muharini et al. (2017). Plant organs: A, aerial parts; L, leaves; F, fruits; S, seeds; R, roots; T, twigs.*

**FIGURE 3:**
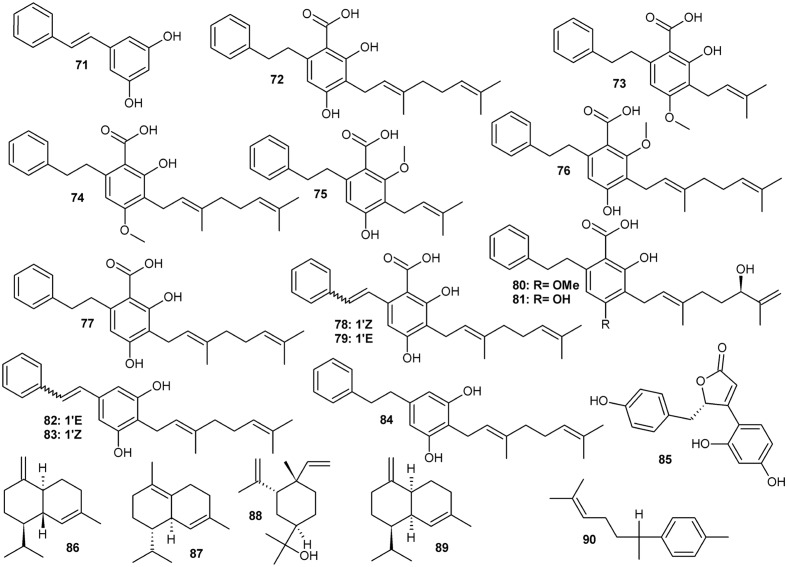
Stilbenoids.

Prenylated stilbenoids (**Figure 3** and **Table 3**) are the second important group of phenolic compounds from *Amorpha*. Several previously unreported derivatives of dihydrostilbene (with various substitutions of the bibenzyl skeleton – depicted in **Figure 3**) have recently been isolated and received a lot of attention as potent antidiabetic agents (via binding to PPARγ, see section on pharmacology) (Weidner et al., 2012; Wang et al., 2014; Fuhr et al., 2015). Further, other phenylpropanoids have been identified as plant constituents along with volatile terpenoids and fatty oils. Interestingly, some of the compounds detected or even isolated from this plant are quite rare or even, at the moment, unique in *A. fruticosa* and have not yet been detected in any other plant species. For example, in their recent paper Muharini et al. (2017) report the isolation and structural elucidation of 14 new compounds, which are mainly rotenoids and geranyl-isoflavones, along with 40 known compounds.

**Table 3:** Stilbenoid compounds, puerarol, and examples of major essential oil constituents from various parts of *Amorpha fruticosa*.

				Contents or obtained
Compound		CAS Registry Number	Plant material	amount mg/g dry weight
**Stilbenoids**				
71	Pinosylvin	22139-77-1	L	n.a
72	2-Carboxy-3,5-dihydroxy-4-geranylbibenzyl		F	2.7
73	Amorfrutin A	80489-90-3	F	1.22
74	Amorfrutin B	78916-42-4	F, S	1.58, 0.38
75	2-Carboxy-3-methoxy-5-hydroxy-4-prenylbibenzyl	Not assigned	L, T	n.a
76	2-Carboxy-5-hydroxy-3-methoxy-4-geranylbibenzyl	Not assigned	L, T	n.a.
77	2-Carboxy-3,5-dihydroxy-4-geranylbibenzyl	73436-04-1	L, T	0.018
78	2-[(Z)-styryl]-5-geranylresorcin-1-carboxylic acid	Not assigned	F	n.a
79	2-[(E)-styryl]-5-geranylresorcin-1-carboxylic acid	Not assigned	F	n.a.
80	Amorfrutin D	Not assigned	F	0.04
81	4-*O*-Demethylamorfrutin D	Not assigned	F	0.05
82	Amorphastilbol	72165-33-4	L, T, F	n.a
83	2-Geranyl-5-[(Z)-styryl]resorcin	Not assigned	F	0.036
84	2-Geranyl-5-(2-phenylethyl) resorcin	Not assigned	F	0.007
**Other**				
85	(+) Puerol A	1803270-52-1		0.036
**Major essential oil constituents**				Contents in essential oil %
86	γ-Cadinene, (sesquiterpene)	39029-41-9	F	3-11%
87	Δ-Cadinene, (sesquiterpene)	483-76-1	F	5.7-13-18%
88	β-Elemol	32142-08-8	L, flowers	29.4%, 0.14%
89	(-)γ-Amorphene	6980-46-7	F	1.3-4.6%
90	ar-Curcumene	644-30-4	F	3.0-18.7%

*Stilbenoids and puerol according to Kemal et al. (1979), Mitscher et al. (1981, 1985), Dat et al. (2008), Chen et al. (2015), Muharini et al. (2017), essential oil according to Motl et al. (1966), Georgiev et al. (2000), Lis and Góra (2001), Ivanescu et al. (2014). Plant organs: A, aerial parts; L, leaves; F, fruits; S, seeds; R, roots; T, twigs.*

## Phytochemical Constituents of *Amorpha fruticosa*: Isoflavonoids (Including Rotenoids)

The history of *A. fruticosa* as a subject of interest for phytochemists can be traced back to the beginning of the 20th century. The first pure isoflavonoid to be isolated from *A. fruticosa* was the hydroxyrotenone glycoside amorphin (**21**), which was isolated along with its aglycone back in the 1940s (Acree et al., 1943). Since then, more than fifty different compounds belonging to the rotenoid group have been identified in *A. fruticosa* (Hegnauer, 2001; Kim et al., 2011; Muharini et al., 2017).

A wealth of phytochemical information about phytochemicals in various plant parts of *A. fruticosa* is available from scientists from Uzbekistan, who published their results in the 1960s and 1970s of the last century. They report isolation of rotenoid glycosides and aglycones, such as amorphigenin (**1**), amorphin (**21** – arabinoglucoside of **1**) and their dehydro- and hydroxy- analogs (Kondratenko et al., 1967; Kasymov et al., 1968, 1969, 1972; Genkina et al., 1971; Kadyrova et al., 1973; Khodzhaev et al., 1982).

For isolation of the major glycoside – amorphin (also known as fruticin, frutitsin, amorphigenin-*O*-vicianoside, where ‘vicianoside’ refers to α-L-arabinopyranosyl-1→6-*O*-β-D-glucopyranoside), simple solvent extraction and subsequent crystallization from the washed precipitate is reported as sufficient to isolate most of the compound contained in the seeds (about 1.5%) and recrystallization yields high purity amorphin corresponding to 0.7% dry mass of the seeds. Subsequently, a series of papers on isolation of the same and some new rotenoids followed, usually applying conventional column chromatography on various stationary phases complemented with preparative or semi-preparative HPLC (Konoshima et al., 1993; Terada et al., 1993; Lee et al., 2006a,b; Kim et al., 2011; Wu et al., 2015; Muharini et al., 2017).

The isolates were mostly various analogs of rotenone or amorphigenin as well as spironone type rotenoids, the backbones of which differ in the configuration of oxygen B and E-rings (**Figure 1**) (Hegnauer, 2001). Several glycosides of the above were also obtained, usually of D-glucose and L-arabinose. One of these compounds was isolated from fruits by Diao et al. (2009) and identified as a new, apparently an amorphigenin-*O*- β-D-glucopyranosyl-α-D-arabinopyranoside. The only difference between this new compound and the major compound amorphin (amorphigenin-*O*-β-D-glucopyranosyl-α-L-arabinopyranoside) is a different stereochemistry of the arabinose-moiety. This compound’s identity would have to be confirmed.

Rotenoid aglycons show complex stereochemistry and interestingly the hitherto isolated compounds are often single stereoisomers (for example compounds **6/9/10**, **2/12**, or **21/22**). The stereochemistry of these compounds should be further explored for its influence on pharmacological properties. Moreover, stereospecificity (or its absence) of biosynthetic routes (enzymes) would be important to understand how the proportions between individual compounds are regulated on a quantitative level.

Several isoflavonoid glycosides and aglycones were isolated along with rotenoids. From the bark, four 7-*O*-β-D-glucopyranosides were obtained with the following aglycones: 4′-methoxyisoflavone, 3′-hydroxy-4′-methoxyisoflavone, 3′,5-dihydroxy-4′-methoxyisoflavone and 4′,6-dimethoxyisoflavone (Lee et al., 2006a). In seeds, different aglycons such as afrormosin (**39**), 7,2′,4′,5′-tetramethoxyisoflavone (**40**), 8-methylretusin (**41**) isoformononetin (**43**), daidzein (**44**), prunetin (**45**), pratensein (**46**), and glycosides ononin (**42**) and 7-*O*-β-D-glucopyranoside of **47** (**48**) were found (Wu et al., 2016), while in fruits, geranylated aglycones (**49-51**) and a daidzein β-sophoroside (**52**) have been identified (Muharini et al., 2017). In roots, formononetin (**53**), calycosin (**54**) and a rare quinone isoflavonoid – amorphaquinone (**55**) have been observed (Shibata and Shimizu, 1978; Ohyama et al., 1998).

A couple of rotenoids and highly prenylated flavonoids were also obtained from root bark (Rózsa et al., 1982, 1984, 1988; Ohyama et al., 1998; Kim et al., 2011). These are mostly flavanones with two to three isoprenyl chains usually attached to carbons C6, C8 or C5’ and sometimes forming additional oxygen rings with adjacent hydroxyls. Most of these prenylflavanones (compounds **58–68**) were unique for roots and have not been reported to be present in aerial parts of *A. fruticosa* so far.

However, despite numerous reports on isolation and bioactivity of a range of rotenoids, it is difficult to find any comprehensive analytic method that would facilitate uniform, comparative profiling and quantitation of possibly all described compounds from *A. fruticosa*.

Kim et al. (2011) report HPLC analysis of eight isoflavonoids (among which are three rotenoids), which were isolated from a root acetone extract. A triprenylflavanone isoamoritin (**63**) and dalbinol (**6 -** a rotenoid) constituted most of the acetone extract from the root bark. These results suggest that the phytochemical profile of roots is markedly different from leaves or fruits/seeds.

Cui et al. (2016) used routine UV-HPLC on a C18 column for quantitative analysis of 15 phenolic compounds that they had isolated from leaves and compared their content in three samples of *A. fruticosa*. However, among those compounds, only two rotenoids (tephrosin **28** and 6a,12a-dehydrodeguelin **34**) were included, along with seven common flavonoids, two sterols, two isoflavonoids, and two phenol carboxylic acids.

## Phytochemical Constituents of *Amorpha fruticosa*: Stilbenoids

More than 10 stilbene derivatives (**71–84**, **Figure 3** and **Table 3**) have been identified to date in *A. fruticosa*. Depending on saturation of the C-C bond between the two aryls, they either belong to typical stilbenes or, if the two carbon link is saturated, to bibenzyls. The latter group, known as amorfrutins is quite diverse in Amorpha, with a carboxyl moiety attached to the aromatic ring and significant variation of prenylation pattern. Amorfrutins, despite being known as present in *A. fruticosa* already since the 1980s (Mitscher et al., 1981, 1985), only recently received a lot of attention due to discovery of their pharmacological properties.

However, amorfrutins and other stilbenoids bearing a carboxyl group, can be also viewed as derivatives of benzoic acid, thus belonging to the phenol carboxylic acids class. A definite classification of these pharmacologically valuable metabolites would require a sound understanding of the metabolic pathways that contribute to assembly of these compounds in Amorpha. Such a classification has not yet been attained. Elucidation of biosynthetic routes of amorfrutins is therefore eagerly needed and should include specific reactions and enzymes responsible for them as well as genes and their expression control. It is essential for understanding the mechanisms of regulation of their production and accumulation. Putatively, a combination of known pathways is involved in the construction of an amorfrutin (carboxydihydrostilbene) backbone. It could be using salicylic acid as substrate via phenylpropanoid or via isochorismate pathways, with subsequent complex addition of isoprenoid and phenylethanoid side groups. Alternatively and more likely, the biosynthetic route could also proceed via formation of stilbenecarboxylate pattern by a stilbenecarboxylate synthase (STCS) in a similar way as in hortensias or liverworts, where dihydro-4-coumaroyl-CoA is a direct substrate for lunularic acid production and the ring folding proceeds without losing a terminal carboxyl group (Eckermann et al., 2003). Similarly, the dihydrostilbene structure may be formed by bibenzyl synthase (BBS) activity from dihydro-4-coumaric-CoA and three molecules of malonyl-CoA (Preisig-Müller et al., 1997). Both enzymes (STCS and BBS) are related to stilbene synthase (STS) but differ in catalytic specificity (Chong et al., 2009). Another possibility, although less likely, would be a formation of a stilbenoid structure via STS, then hydrogenation of the double bond linking the two aryl rings, followed by carboxylation and other substitutions of the dihydrostilbene backbone.

Among plant sources used for obtaining amorfrutins, *Glycyrrhiza foetida* Desf. roots seem to be more abundant in these metabolites (Weidner et al., 2012). However, Amorpha seeds which contain about 1.5% of total prenylated carboxydihydrostilbenoids could represent a sustainable and easy to process resource for drug leads or phytomedicinal preparations for use in diabetes. An extensive screening of amorfrutin content in various populations and investigation of factors that regulate their accumulation to constant quantities would be beneficial for evaluating the possibility of using this plant as alternate industrial crop.

A report by Chen et al. (2016) demonstrates significant variations in amorfrutin content between samples from different locations in China. Using HPLC, it was shown that *A. fruticosa* seeds contain between 2.6 mg/g up and 15.6 mg/g of amorfrutin A, B, and C in sum. The same team has also established a preparative method using HSCCC separation to recover a total of around 100 mg high purity amorfrutins from 100 g of seeds (Chen et al., 2015). These results suggest *A. fruticosa* seeds as feasible material for obtaining larger amounts of amorfrutins for further investigations.

It is noteworthy that a reliable universal method for determination of possibly all potentially bioactive metabolites, including rotenoids and prenyl-stilbenoids is still not available and is particularly awaited.

## Phytochemical Constituents of *Amorpha fruticosa*: Volatile Constituents of Essential Oil

Both leaves and the untypical fruits with one seeded pods contain a significant volatile fraction, consisting mainly of sesquiterpenoids, both hydrocarbons and oxygenated forms. Depending on the precise location where the plant material has been collected, various compounds are reported as major (Georgiev et al., 2000; Lis and Góra, 2001). Some of the major sesquiterpenes are listed in **Table 3**, and shown in **Figure 3**.

For example, in essential oil from fruit, γ-, and δ-cadinenes (**86, 87**), β-elemol (**88**) and β-caryophyllene predominated in samples from Bulgaria and Romanian Moldova (Stoyanova et al., 2003; Ivanescu et al., 2014), while γ-muurolene and ar-curcumene (**90**) or α-pinene and myrcene were most abundant in samples from Poland (Lis and Góra, 2001). The leaf oil contains mainly α-eudesmol, (E)-β-ocimene, and α-pinene (Lis and Góra, 2001). An uncommon cadinene stereoisomer named (-)γ-amorphene (**89**) was isolated and identified from samples collected in Ukraine. This compound, although characteristic to this plant, has been detected in small (ca.5–6% of total oil) amounts only and accompanies other cadinene-type stereoisomers (Motl et al., 1966; Georgiev et al., 2000).

The yield of essential oil from leaves reaches 0.23% and from fruits as much as 1.1%. The essential oil has a pleasant scent due to the mixture of highly aromatic terpenoids. However, its inconsistent composition is a limitation for its use in aromatherapy and as a medication. More research is needed to elucidate the mechanisms of this diversity and regulation of volatile organic compounds production and accumulation.

Volatile organic compounds in headspace as well as ultrasonic extracts of unifloral *A. fruticosa* bee honey were also analyzed and a number of aroma compounds identified, none of which was, however, unique for Amorpha honey. Also 2-phenylethanol, linalool oxide and a few benzoic acid esters were present as major compounds. However, the significance of this study to extrapolate to nectar composition is somewhat limited, since in honey most of the constituents could form during processing by bees or during storage and therefore, do not necessarily reflect the nectar composition (Jerković, 2009).

## Phytochemical Constituents of *Amorpha fruticosa*: Fatty Acids and Carbohydrates

In fatty oil extracted by supercritical CO_2_ fluid from *A. fruticosa* seeds, linoleic (66.4%) and oleic acid (11.95%) predominated whereas stearic and palmitic acid were the only saturated ones present in significant amounts (both 7%). None of the remaining 18 detected acids exceeded 1% and the extraction efficiency was 7.5% of the seeds mass (Wei et al., 2016).

A single report on polysaccharides from seeds of *A. fruticosa* indicates 2.6% content of galactomannane consisting of D-mannose and D-galactose in a ratio of 1.63:1, in which the main chain is built of 1→4-β-D-mannopyranose monomers with single α-D-galactopyranose residues attached (Mestechkina et al., 1998).

## Pharmacological Activity of *Amorpha fruticosa*: Antidiabetic Properties

The most studied pharmacological effect of *A. fruticosa* is its antidiabetic effect. In the recent years, metabolic diseases such as diabetes type 2 have developed to the scale of a global epidemic (Smyth and Heron, 2006). The nuclear receptor PPARγ is a key regulator of lipid and glucose metabolism and has proved to be a viable therapeutic molecular target. However, although highly effective, currently used PPARγ-targeting drugs applied in clinics are having unwanted side effects, and this is why safer PPARy-targeting drugs are being sought. After food intake, PPARγ activity is modulated by binding of lipid food constituents, which can act as PPARγ ligands, among others unsaturated fatty acids, overall resulting in changes of the expression of a large number of metabolism-related genes (Weidner et al., 2012). Interestingly, many compounds derived from medicinal foods or dietary spices also display PPARγ-activating properties (Atanasov et al., 2013; Pferschy-Wenzig et al., 2014; Wang et al., 2014). To identify new food-derived PPARγ-activating compounds, researchers from Germany (Weidner et al., 2012) examined a natural products library of approximately 8000 molecules derived largely from edible biomaterials. As a result of this focused effort, amorfrutins were found, a structurally new class of highly potent PPARγ ligands. Amorfrutins have been present in this compound library focused on edible materials since these compounds are present in the fruits of *Amorpha fruticosa*, which are used as an ingredient in some condiments. The PPARγ receptor binding affinity constants of studied amorfrutins ranged from 236 to 354 nM, revealing a several-fold greater potency than a synthetic clinically used drug (pioglitazone) that was used as a positive control. Some of the studied amorfrutins were also able to act as less potent activators of other PPAR isoforms, PPARα and PPARβ/δ (Weidner et al., 2012). In the same study, the researchers also tested for *in vivo* effectiveness a chemically synthesized amorfrutin 1 using insulin resistant high-fat diet-induced obesity (DIO) mice model. Application of amorfrutin 1 for 23 days, resulted in significant reduction of insulin resistance, similar to the reduction observed with the clinically used drug rosiglitazone. Glucose tolerance and insulin sensitivity were also enhanced, as revealed by oral glucose tolerance and intraperitoneal insulin sensitivity tests. Plasma triglycerides, insulin, free fatty acids, and glucose were reduced to an extent comparable to the positive control, rosiglitazone. Interestingly, in contrast to rosiglitazone, amorfrutin 1 also significantly reduced weight gain. Authors also studied the antidiabetic effects of amorfrutin 1 in leptin receptor-deficient db/db mice, and found that the compound was able to decrease plasma insulin levels even more potently than rosiglitazone, and also prevented the deterioration of pancreatic functionality. In summary, the results from the investigation of Weidner et al. (2012), suggested that amorfrutins are selective PPARγ modulators with anti-diabetic properties and overall have a more favorable bioactivity profile than the synthetic clinically used PPARγ agonists from the thiazolidinedione class.

Moreover, amorfrutins, isolated from *A. fruticosa*, are shown to act as inhibitors of the nuclear transcription factor-κB (NF-κB) signaling pathway by blocking NF-κB/DNA binding (Dat et al., 2008). This bioactivity is of high relevance, because NF-κB is a key regulator of inflammation, and the pro-inflammatory NF-κB activation contributes to the pathogenesis of diabetes, which overall might suggest that anti-inflammatory effects of amorfrutins may potentially be also implied in their antidiabetic action.

In addition to amorfrutins other compounds present in *A. fruticosa* have been evaluated for their potential as antidiabetic agents. In this context, the effects of amorphastilbol (APH), another constituent from *A. fruticosa*, was studied *in vitro* with 3T3-L1 adipocytes, as well as *in vivo* with db/db and high-fat-diet (HFD) mice (Lee et al., 2013, 2015). It was observed that the compound is able to stimulate the transcriptional activities of PPARγ and PPARα, resulting in beneficial effects on the metabolism of lipids and glucose without significant side effects that are notoriously associated with stimulation of the PPAR receptors, such as weight gain or hepatomegaly. APH was also able to improve insulin sensitivity via inhibition of protein tyrosine phosphatase 1B (Lee et al., 2015).

Also 5,7-dihydroxy-6-geranylflavanone, another constituent from *A. fruticosa*, is reported to act as PPARα/PPARγ dual activator, being able to stimulate adipocyte differentiation of 3T3-L1 cells (Lee et al., 2016).

## Pharmacological Activity of *Amorpha fruticosa*: Anti-Inflammatory And Anti-Tumor Activities

The NF-κB pathway controls many physiological processes, including apoptosis, inflammatory responses, and angiogenesis. Amorfrutin A from the fruits of *A. fruticosa* was identified as a NF-κB inhibitor (Shi et al., 2014) suppressing TNF-α-activated IκBα degradation, NF-κB p65 subunit nuclear translocation, and the NF-κB DNA-binding. Moreover, it potentiated TNF-α-induced (NF-κB signaling mediated) apoptosis. This activity pattern suggests the compound as an anti-inflammatory lead and might help to rationalize some of the uses of *A. fruticosa* in traditional herbal medicine (Shi et al., 2014).

As discussed above, amorfrutins were shown to be potently acting ligands of PPARγ. Fuhr et al. (2015) studied PPARγ-related anti-inflammatory effects of amorfrutins colon cells. Indeed it was observed that amorfrutin A reduces the expression of several inflammation mediators, at least in part due to activation of PPARγ. This activity pattern suggests amorfrutins are promising molecules with potential application in the treatment of inflammatory bowel disease or other inflammation-associated disorders.

## Pharmacological Activity of *Amorpha fruticosa*: Cytotoxicity

The toxic potential of extracts and biologically active substances (BAS) from *Amorpha fruticosa* was evaluated principally by *in vitro* methods. Li et al. (1993) isolated eight cytotoxic compounds from a CHCl_3_ extract of *A. fruticosa*. One of these compounds, 6′-*O*-D-beta-glucopyranosyldalpanol, was described as a new cytotoxic rotenoid. Another known rotenoid, 12 alpha beta-hydroxyamorphigenin, was shown to exhibit extremely potent cytotoxicity (ED50 < 0.001 μg/ml) in six neoplastic cell lines.

As a part of screening studies for cytotoxic agents (anti-tumor-promoters), six North American plants belonging to the *Amorpha* genus were tested using an *in vitro* assay (Konoshima et al., 1993). Authors found that *A. fruticosa* exhibited strong inhibitory effects on Epstein-Barr virus early antigen (EBA-EA) activation induced by 12-*O*-tetradecanoylphorbol-13-acetate. Six rotenoids, were isolated from the leaves of *A. fruticosa*, among which were amorphispironone and tephrosin. Apart from inhibition of EBA-EA activation, these compounds also displayed anti-tumor effects on mouse skin *in vivo*.

Eight rotenoid glycosides, including four new rotenoid glycosides, namely amorphasides, along with four known ones, all of them isolated from the seeds of *A. fruticosa* were evaluated for *in vitro* cytotoxicity against the MCF-7 and HCT-116 tumor cell lines (Wu et al., 2015). Three of the investigated substances had no effect on cell proliferation for the two cell lines even applied at 50 μM. Three other compounds had selective cytotoxicity against MCF-7. The other two compounds both displayed cytotoxicity to the two cell lines with IC_50_ values of less than 2.00 μM, which was even superior to the effect of cisplatin (Wu et al., 2015). Also rotenoids and their derivatives, being present as dominant substances in the crude extract of fruits from *A. fruticosa*, displayed significant cytotoxicity when tested against the L5178Y mouse lymphoma cell line in concentrations between 0.2 and 10.2 μM (Muharini et al., 2017).

## Pharmacological Activity of *Amorpha fruticosa*: Antimicrobial (Antibacterial and Antifungal) Activity and Wound Healing Effects

Microbial pathogens are known to delay wound healing (Macri and Clark, 2009). *A. fruticosa* leaves and fruits have been used for treating wounds in traditional medicine. Interestingly, some constituents of *A. fruticosa* have antimicrobial potentials, thus promoting wound healing (Qu et al., 2013). Some of the compounds stimulated proliferation and migration of fibroblasts, a key cell type involved in wound healing (Hinz, 2010; Qu et al., 2013), and also collagen synthesis was improved upon topical application of ointment containing some of the compounds isolated from *A. fruticosa* (10% w/w). In the same study, the inhibitory effects of seven compounds isolated from the fruits of *A. fruticosa* on some Gram positive and Gram negative bacteria growth were also evaluated. Three of the seven isolated compounds, including the two that also promoted wound healing showed effective Minimal Inhibitory Concentrations (MIC) and Minimum Bactericidal Concentrations (MBC) of compounds against *Bacillus subtilis, Staphylococcus aureus, Pseudomonas aeruginosa, Bacillus cerculences, Escherichia coli*, and *Klebsiella pneumonia* in a range between 0.1 and 0.2 mg/ml. These results suggest suggested that the three identified substances with rotenoid structures might be an important contributor to the wound promoting effects of *A. fruticosa*.

Antimicrobial activity of seeds from *A. fruticosa* from the Mississippi river basin was assessed as moderately effective, mainly against *S. aureus* (Borchardt et al., 2009). Essential oils from *A. fruticosa* fruits were found to exhibit moderate antimicrobial activity against Gram-positive bacteria (Ivanescu et al., 2014). In another study to elucidate antibacterial properties of *A. fruticosa*, several geranylated bibenzyl compounds extracted from *A. fruticosa* fruits, exhibited significant antibacterial activity against Gram-positive bacteria (Muharini et al., 2017).

Four flavanones and three rotenoids obtained via activity-guided isolation from an acetone extract of *A. fruticosa* roots (amoradicin, amorisin, isoamoritin, amoricin, amorphigeni, dalbinol, and 6-ketodehydroamorphigenin) showed strong neuraminidase inhibition *in vitro*. Some pathogens, e.g., *Haemophilus influenza* (Greiner et al., 2004) or *Streptococcus pneumoniae* (Donlan et al., 2004), require neuraminidase in order to proliferate. In particular, one of the compounds, amorisin, exhibited more potent inhibition (IC_50_ = 0.12 μM) than the reference compound, quercetin (Kim et al., 2011). To challenge their promising *in vitro* results, the authors then tested the isolated compounds against living bacteria in a straightforward biofilm-assay, since it is known that neuraminidase plays an important role in biofilm formation (Soong et al., 2006). The authors were able to show that two of the seven compounds were able to inhibit biofilm formation in micromolar concentrations without showing toxicity to the cells.

## Pharmacological Activity of *Amorpha fruticosa*: Antioxidant and Acetylcholinesterase Inhibition Properties

DPPH radical scavenging activity of *A. fruticosa* seeds from the Mississippi river basin was reported (Borchardt et al., 2009). This effect was also supported by another study, where in order to discover new natural sources for treatment of neurodegenerative disorders, methanol extracts from leaves and fruits of *A. fruticosa* were investigated for their antioxidant and acetylcholinesterase inhibitory activity (Zheleva-Dimitrova, 2013). While both methanolic extracts showed activity, the fruit extract demonstrated higher antioxidant activity in two different assays (DPPH radical scavenging activity, ABTS radical scavenging assay), while having a lower number of total polyphenols. The determined IC_50_-values for antioxidant activity were in the low μg/ml range and superior to the positive control butylated hydroxytoluene (BHT), but acetylcholinesterase inhibitory activity of both extracts was lower than that of the positive control galantamine hydrobromide. In conclusion, *A. fruticosa* could be a useful source for agents useful for the therapy of free radical production-associated pathologies.

## Pharmacological Activity of *Amorpha fruticosa*: Hepatoprotective Effects

Diao et al. (2009) studied the protective potential of a new amorphigenin glycoside (**Table 1** – compound **22**, **Figure 1**) against acetaminophen-induced hepatotoxicity by measuring relevant biochemical markers of hepatic injury such as alanine aminotransferase (ALT), aspartate aminotransferase (AST) and hepatic glycogen. It was found this compound could protect liver from hepatotoxicity induced by acetaminophen (AAP). Further hepatoprotective properties of *A. fruticosa*, independent of amorphigenin glycoside, might be due to the described above antioxidant effects of this plant.

## Pharmacological Activity of *Amorpha fruticosa*: Osteoclast Inhibitory Effect

Among other rotenoids, amorphigenin was isolated from the leaves of *A. fruticosa* and was shown to have broad anti-proliferative and anti-tumor effects in diverse cell models (Kim et al., 2010). Amorphigenin abolishes RANKL-induced osteoclast differentiation of bone marrow-derived macrophages by down-regulation of c-fos and NFATc1, without affecting cell viability (Kim et al., 2010). The compound also abolishes RANKL-induced p38 and NF-κB activation. Moreover, amorphigenin protected against LPS-induced bone loss as revealed by micro-CT analysis of the femurs in mice. Overall these results suggest that amorphigenin has a therapeutic potential in the context of inflammation-induced bone loss.

## Pharmacological Activity of *Amorpha fruticosa*: Insect Repellent and Insecticidal Activity

Studies from the 1940s showed that extracts of *A. fruticosa* have repellent and insecticidal activity against diverse insect species (Brett, 1946a,b). The acetone extract of *A. fruticosa* seeds had stronger insecticidal activity against *Aedes aegypti* larvae than 1% pure rotenone (Brett, 1946b). Ethanol extract of *A. fruticosa* seeds has demonstrated good contact effects and antifeedant activity against *Schizaphis graminum* (Ji et al., 2011). Larvicidal activity of extracts and of amorphigenin, isolated from *A. fruticosa* seeds against early fourth-instar larvae of the mosquito *Culex pipiens pallens* was also investigated (Liang et al., 2015). It was found that amorphigenin decreased mitochondrial complex I activities and the protein content. The authors concluded that amorphigenin could be a good candidate for a natural, effective and safe agent that might be used in population control of *C. pipiens pallens.*

In another work, the inhibitory action of amorphigenin against the mitochondrial complex I of *C. pipiens pallens* was studied in comparison to rotenone (Mingshan et al., 2015). It was observed that both compounds abolish mitochondrial complex I activity *in vitro* and *in vivo.* Mixed-I type inhibition of the mitochondrial complex I of *C. pipiens pallens* was observed, suggesting that both compounds are able to bind not just the enzyme but also the enzyme-substrate complex.

## Pharmacological Activity of *Amorpha fruticosa*: Toxicity

All studies listed above show that BASs isolated from *A. fruticosa* are toxic for some microorganisms and some insects. In fact, this selective toxicity might be helpful for macro-organisms and human beings. Until now there are no published data about human toxicity of *A. fruticosa.* On the contrary, *A. fruticosa* and compounds isolated from it showed many positive and useful effects for humans.

## Conclusion

**FIGURE 4:**
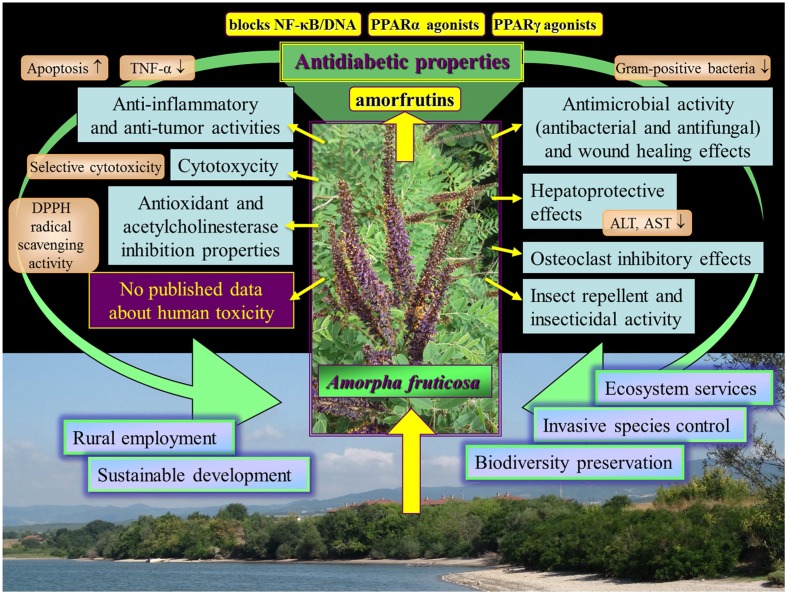
Outline of the different bioeffects of *Amorpha fruticosa* reviewed in this work.

The different effects of *Amorpha fruticosa* reviewed in this work are outlined in **Figure 4**. The potential of *Amorpha fruticosa* against diabetes and metabolic disease is promising and deserves further investigation. The toxicity review in relation to safety application revealed that until now there are no published data about human toxicity of *A. fruticosa.* On the contrary *A. fruticosa* and compounds isolated from it showed many positive and useful effects for humans. This aggressive invasive species provides endless, cheap resource which can be utilized for remedial purposes. A vast use of *A. fruticosa* substances in the future might contribute to resolve problems associated with this aggressive invasive species in the natural habitats in many European countries.

## Author Contributions

EK, AM, DW, RS, ZN, and AA wrote the first draft of the manuscript. CM, AM, and SN revised and improved the first draft. All authors have seen and agreed on the finally submitted version of the manuscript.

## Conflict of Interest Statement

The authors declare that the research was conducted in the absence of any commercial or financial relationships that could be construed as a potential conflict of interest.
